# Promoting mother‐infant relationships and underlying neural correlates: Results from a randomized controlled trial of a home‐visiting program for adolescent mothers in Brazil

**DOI:** 10.1111/desc.13113

**Published:** 2021-04-12

**Authors:** Fernanda Speggiorin Pereira Alarcão, Elizabeth Shephard, Daniel Fatori, Renata Amável, Anna Chiesa, Lislaine Fracolli, Alicia Matijasevich, Helena Brentani, Charles A. Nelson, James Leckman, Eurípedes Constantino Miguel, Guilherme V. Polanczyk

**Affiliations:** ^1^ Department of Psychiatry Faculdade de Medicina FMUSP, Universidade de São Paulo São Paulo SP Brazil; ^2^ Institute of Psychiatry Psychology & Neuroscience King's College London London UK; ^3^ School of Nursing Faculdade de Medicina FMUSP, Universidade de São Paulo São Paulo Brazil; ^4^ Department of Preventive Medicine Faculdade de Medicina FMUSP, Universidade de São Paulo São Paulo SP Brazil; ^5^ Laboratories of Cognitive Neuroscience Division of Developmental Medicine Boston Children's Hospital Harvard Medical School Boston USA; ^6^ Harvard Graduate School of Education Harvard University Boston USA; ^7^ Yale Child Study Center Yale University School of Medicine New Haven USA

**Keywords:** EEG, home‐visiting intervention, infant social development, maternal care competencies, mother‐infant attachment

## Abstract

Poverty and teenage pregnancy are common in low‐and‐middle‐income countries and can impede the development of healthy parent‐child relationships. This study aimed to test whether a home‐visiting intervention could improve early attachment relationships between adolescent mothers and their infants living in poverty in Brazil. Analyses were conducted on secondary outcomes from a randomized controlled trial (NCT0280718) testing the efficacy of a home‐visiting program, *Primeiros Laços*, on adolescent mothers’ health and parenting skills and their infants’ development. Pregnant youth were randomized to intervention (*n *= 40) or care‐as‐usual (CAU, *n *= 40) from the first trimester of pregnancy until infants were aged 24 months. Mother‐infant attachment was coded during a mother‐infant interaction when the infants were aged 12 months. Electrophysiological correlates of social processing (mean amplitude of the Nc component) were measured while infants viewed facial images of the mother and a stranger at age 6 months. Infants in the intervention group were more securely attached and more involved with their mothers than those receiving CAU at 12 months. Smaller Nc amplitudes to the mother's face at 6 months were associated with better social behavior at 12 months. Our findings indicate that the *Primeiros Laços Program* is effective in enhancing the development of mother‐infant attachment.

## 1 INTRODUCTION

Worldwide, children face adversities such as displacement, violence, abuse and poverty, which increase risk for developmental impairments with lifelong consequences (Clark et al., 2020). It is estimated that, globally, over 250 million children (43%) aged 0–5 years do not reach their developmental potential due to exposure to environmental, biological, and psychosocial risk‐factors in low‐and‐middle‐income countries (LMICs) (Black et al., 2017). Improving these children's lives is currently a global priority (Clark et al., 2020). There are 10.3 million children aged 0–3 years in Brazil, and the families of almost half of these live on less than $578 per month (Pnad, 2015). Poverty predicts other risk‐factors for impaired child development, including adolescent parenthood (Lund et al., 2018).

Adolescent parenthood remains a common social problem, particularly in LMICs (World Health Organisation, 2020). Brazil has one of the highest rates of adolescent pregnancy: one in five babies is born to a teenage mother, and three in five of those mothers do not work or study (UNFPA, 2017). Parenthood disrupts typical emotional and cognitive development in adolescence, which can impair the emergence of reflective and responsive parenting skills necessary to foster secure parent‐infant relationships (Flaherty & Sadler, 2011). Being born to an adolescent mother is associated with lower cognitive ability and elevated behavioral problems in childhood (Lee et al., 2020; Morinis et al., 2013), which persist into adolescence and adulthood resulting in increased systemic and mental health disorders, welfare dependency, lower income, and higher criminality (Jaffee et al., 2001; Shaw et al., 2006). These outcomes may occur because of the lower quality of maternal care that adolescent mothers provide, including lower tendency to touch, call, smile at, and accept their child's emotions (Firk et al., 2018). Adolescent mothers are less sensitive to their child's needs, provide less verbal stimulation, and play less with their babies (Crugnola et al., 2014). These responsive behaviors from the mother are essential because they play a causal role in shaping attachment (Zeegers et al., 2017). Secure parent‐child attachments help children to regulate emotions, develop autonomy and resilience, improve their cognitive development, and engage in positive and cooperative relationships (Groh et al., 2017). However, insecure and disorganized attachment places children at risk of problematic behaviors and psychopathology (Ainsworth et al., 1978; Groh et al., 2017) and is a significant contributor to public costs in adolescents with antisocial behavior (Bachmann et al., 2019). The estimated rate of secure attachment in the general population is 59%–67% (Bakerman‐Kranenburg & van IJzendoorn, 2009; Lewis‐Morrarty et al., 2014), while rates of secure attachment in infants of adolescent mothers are ∼33% (Moran et al., 2008).

Home‐visiting programs (HPVs) designed to meet families' needs with young children lead to positive effects on maternal and child health, child development, responsive parenting, secure attachment, and maltreatment rates (Filene et al., 2016). The most well‐established HVPs (e.g., Early Head Start, Nurse‐Family Partnership) support infant‐parent relationships and are grounded in attachment theory, given the crucial role of early parent‐infant attachment in development (Mountain et al, 2017). HVPs have been used with adolescent mothers in high‐income countries and showed improved parenting knowledge and behaviors, including increased maternal sensitivity and stimulation of the baby as well as improved socio‐emotional development and reduced externalizing behavior problems in early‐childhood in offspring (Barlow et al., 2015; Guttentag et al., 2014). HVPs have also shown positive effects on maternal parenting behaviors and offspring development in vulnerable adult mothers in LMICs, including India (Andrew et al., 2020), South Africa (Christodoulou et al., 2019), Rwanda (Betancourt et al., 2020) and Brazil (Gonçalves et al., 2019).

**Research highlights**

- Teenage pregnancy is common in low‐and‐middle‐income countries (LMICs)
- Adolescent mothers often have less secure relationships with their babies
- A home‐visiting intervention improved attachment relationships in this population
- Better attachment was associated with enhanced neural processing of social stimuli

Yet, despite the potential to improve maternal parenting behavior and infant development, to our knowledge no published work has assessed the efficacy of HVPs developed specifically for adolescent mothers in LMICs. We therefore conducted a randomized controlled trial (RCT) of an HVP designed for adolescent mothers and their infants living in poverty in Brazil (*Primeiros Laços*). We created the program based on the Nurse‐Family Partnership and Minding the Baby, two programs with a solid conceptual basis and empirically‐supported efficacy (Olds, 2006; Slade et al., 2005), but adapted for the socio‐cultural characteristics and health system organization in Brazil (Fracolli et al., 2018; Pinheiro et al., 2018). The primary aim of the trial was to investigate the efficacy of the HVP on infants’ cognitive, motor and language development, and also the effects of the intervention on important secondary outcomes including maternal well‐being, parenting behaviors, mother‐infant attachment relationships and neurobiological mechanisms involved in the intervention (clinicaltrials.gov NCT02807818). In the current analysis, we focused on two of these secondary outcomes: (1) mother‐infant attachment relationships, and (2) neural correlates of infants’ social development, as well as the relationship between these outcomes.

Mother‐infant attachment relationships were measured during a mother‐infant interaction episode when the infants were aged 12 months. Neural correlates of social development were measured with a component of the event‐related potential (ERP), the Nc, derived from electroencephalography (EEG) recorded while the infants viewed images of their mother's face and a stranger's face when they were aged 6 months. The Nc reflects attentional and memory processes and is sensitive to social information; in the first year of life, the Nc is larger (i.e. has greater amplitude) when viewing the mother than a stranger, reflecting enhanced attentional allocation to the socially‐salient mother (de Haan & Nelson, 1997). This effect reverses from the second year of life, coinciding with the emergence of more exploratory social behaviors with individuals other than the mother (Carver et al., 2003). The Nc to mother/stranger faces appears to be particularly sensitive to children's social behavior and relationships with their mothers. One study reported that, compared to securely‐attached preschoolers, insecurely‐attached preschoolers showed larger Nc amplitudes when viewing their mother's face, suggestive of greater attentional allocation to the mother in children with less stable maternal relationships (Kungl et al., 2017). Similarly, another study found that infants who showed more social engagement behaviors with the mother following a brief separation showed smaller Nc amplitudes to the mother's face than a stranger's face, interpreted as reflecting less attentional allocation to the mother in infants who held a stable representation of the mother as being someone to whom the infants could direct their social‐engagement behaviors while exploring their environment (Swingler et al., 2007).

We hypothesized that (1) mother‐infant dyads who received the intervention would show stronger attachment relationships at 12 months than those receiving care‐as‐usual, (2) consistent with previous work investigating the Nc in the first year of life (de Haan & Nelson, 1997), at age 6 months the infants in the intervention group would show more differentiated neural responses to mother and stranger face stimuli, as indicated by larger Nc to the mother's than stranger's face, compared to infants receiving care‐as‐usual, and (3) more differentiated Nc at 6 months would be associated with better social‐interaction behavior with mothers at 12 months.

## 2 METHODS

### 2.1 Study design and participants

**FIGURE 1:**
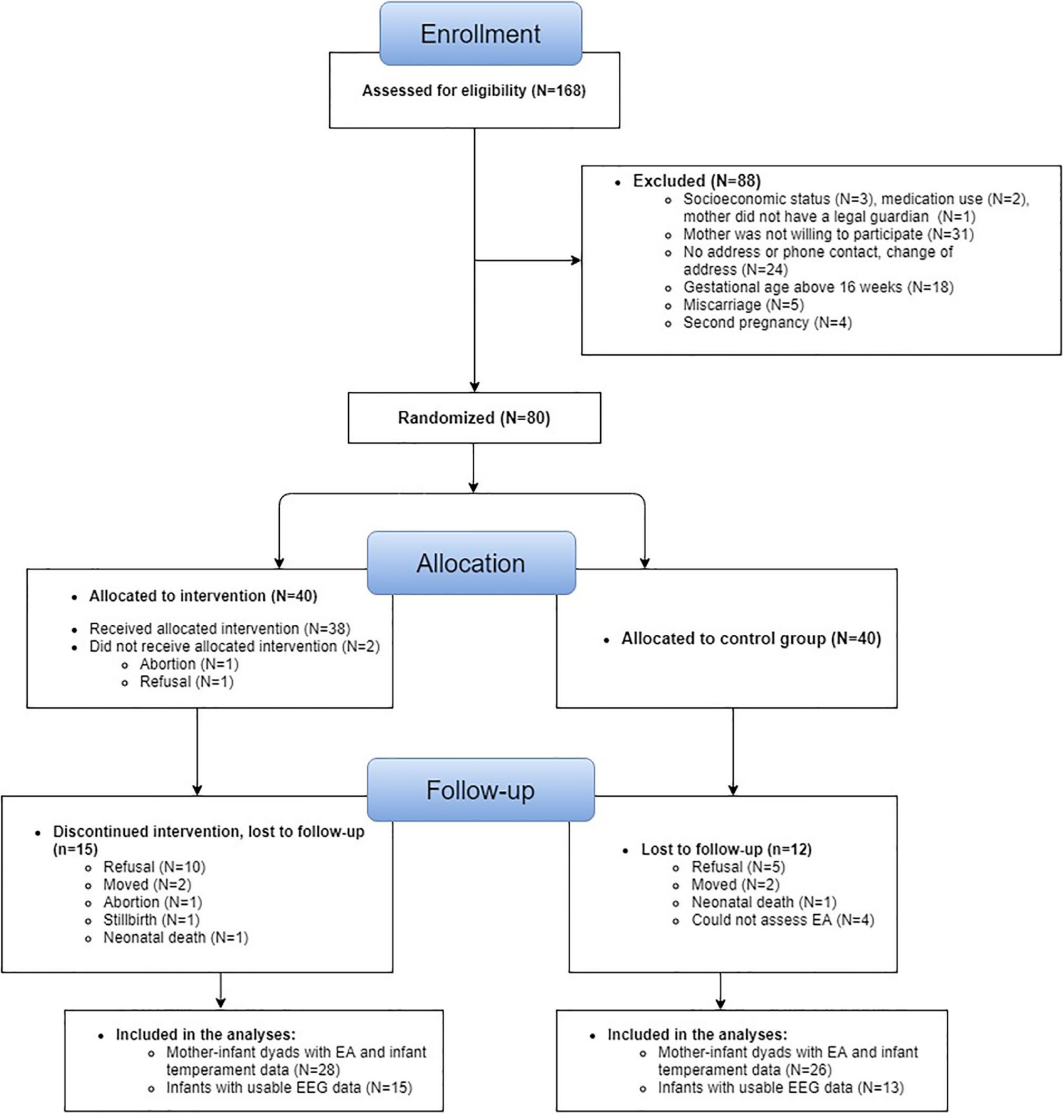
CONSORT diagram of participant enrolment, assessment and randomization

The current report presents analysis of secondary outcome measures collected as part of an RCT (clinicaltrials.gov NCT02807818) conducted to test the efficacy of the *Primeiros Laços* HVP intervention for adolescent mothers and their infants living in deprived urban areas of São Paulo, Brazil. The main findings from the trial are reported elsewhere (Fatori et al., under review) as are analyses of other secondary outcome measures (Fatori et al., 2020; Shephard et al., 2019). Pregnant youth were recruited from primary healthcare units in Western regions of São Paulo between June‐September 2015 and assessed for eligibility criteria. Eligibility criteria were: aged 14–19 years, first pregnancy, 8–16 weeks gestation, low socioeconomic status (classes C/D/E according to the Brazilian classification system, ABEP, 2007), and living in impoverished western regions of the city of São Paulo. These regions are characterized by high rates of urban violence, widespread slums, adverse living conditions, and lower access to medical and public resources. Of 168 pregnant adolescents initially screened, 80 met eligibility criteria and were individually randomized to intervention plus care‐as‐usual (Intervention group, n = 40) or care‐as‐usual alone (CAU group, n = 40) groups (see CONSORT diagram in Figure 1). Randomization was stratified by primary healthcare unit type (with vs. without family health support teams) and grandmother's years of schooling. Group allocation ratio was 1:1. Further details of the RCT can be found elsewhere (https://clinicaltrials.gov/ct2/show/NCT02807818; Fatori et al., 2020; Fatori et al., under review; Shephard et al., 2019). In accordance with the Declaration of Helsinki, written informed consent was obtained from all adolescent mothers and, if aged < 18 years, their parent/guardian. The study was approved by the ethical review boards of the University of São Paulo Medical School (ref: 052/15) and the São Paulo Municipal Health Department.

### 2.2 Intervention

*Primeiros Laços* is an HVP delivered by trained nurses tailored to first‐time pregnant adolescents and their infants, running from the first 16 weeks of pregnancy until the child is aged 24 months. Nurses were specialized in maternal or mental health and were supervised weekly by senior nurses and psychologists. *Primeiros Laços* is based on three theoretical frameworks (*Attachment theory*, Bowlby et al. 1979; *Self‐Efficacy theory*, Bandura, 1977; *Bioecological Development theory*, Bronfenbrenner, 1998) and was structured in five axes (*health care*, *health environment*, *parenting and attachment*, *social and family network*, *life project*) (see eTable for further details). Our team developed the program based on existing HVPs (Olds, 2006; Slade et al., 2005). Each mother‐infant dyad's care plan was carefully designed to strengthen maternal competences for warm and responsive care. A key factor was establishing positive relationships between home‐visitors and the family. During the visits, nurses helped parents develop child‐centered interactions, improve their bond with their infant, reflect on their attachment history and the parenting they received, consider their child as individuals with their own needs, feelings, and thoughts, and improve their parenting skills by modeling. The Visitors encouraged attuned parenting and stimulated sensitive behaviors, such as being attentive to the child's communicative signals or following their lead. Parents were also given support to think reflectively. The frequency of visits was weekly (first/last month of pregnancy/puerperium), biweekly (gestation/2‐20 months of child's age), and monthly (21‐24 months of child's age). Mothers were expected to receive 40–42 visits by the time their infants were aged 12 months and 60–62 visits by the time their infants were 24 months of age. Participants who received the intervention also had access to public health services (care‐as‐usual).

**TABLE 1:** Socio‐demographic characteristics at baseline by intervention and care‐as‐usual (CAU) group

	**CAU N = 40**	**Intervention N = 40**	**Total N = 80**	**Group differences (*p*‐value)**
*Maternal age (mean years (SD))*	17.28 (1.21)	16.95 (1.28)	17.11 (1.25)	0.248
*Mother enrolled in school (N (%))*	19 (47.5%)	16 (40.0%)	35 (43.8%)	0.326
*Maternal education level (N illiterate (%))*	4 (10.0%)	8 (20.0%)	12 (15.0%)	0.210
*Maternal occupation (N working for pay (%))*	10 (25.0%)	5 (12.5%)	15 (18.8%)	0.152
*Grandmother's education level (N illiterate (%)*	21 (52.5%)	16 (40.0%)	37 (46.3%)	0.262
*Family monthly income (N BRL 0–800 (%))*	7 (18.9%)	8 (22.2%)	15 (18.8%)	0.727
*Presence of family food insecurity (N (%))*	13 (32.5%)	21 (52.5%)	34 (42.5%)	0.070
*Family enrolled in a welfare program (N (%))*	11 (27.5%)	10 (25.0%)	21 (26.3%)	0.799
*Number of people living in the residence (mean, SD)*	3.6 (1.4)	3.6 (1.9)	3.6 (1.7)	0.947
*Maternal depression (N with BDI scores in moderate/severe range (%))*	7 (17.5%)	10 (25.0%)	17 (21.3%)	0.586
*Maternal anxiety (N with BAI scores in moderate/severe range (%))*	7 (17.5%)	12 (30.0%0	19 (23.8%)	0.189

Food insecurity was measured with the Brazilian Food Insecurity Scale. BRL = Brazilian reais (R$). BAI & BDI = Beck anxiety and depression inventories; BDI moderate/severe range = scores > 20; BAI moderate/severe range = scores > 16.

### 2.3 Care‐as‐usual (CAU)

Adolescent mothers in the CAU group received standard prenatal and postnatal care‐as‐usual. In Brazil, prenatal and postpartum care is offered by the Unified Health System (SUS) to all Brazilian women. This assistance is based in the municipalities and is carried out by Family Health Care Teams. Prenatal care consists of the following routines: (1) monthly consultations (with nurses or doctors) until 30 weeks of gestation and thereafter fortnightly; (2) multidisciplinary monitoring; (3) referral to specialist if risk detected; (4) mandatory ultrasound examination before 20 weeks and after 26 weeks; (5) serological tests in the first trimester of pregnancy and in the last semester of pregnancy (to check for anemia, syphilis infection, blood glucose, among others). Prenatal care is organized to meet the bio‐psycho‐social demands of women and their families. Upon completing 40 weeks of gestation, the woman is referred to the regional hospital where her delivery will take place. Puerperal assistance consists of home care performed from the 15th day of the baby's birth and then continues with medical consultations in primary health care units (Schirmer, 2000).

### 2.4 Measures

#### 2.4.1 Baseline assessments (8‐16 weeks gestation)

Sociodemographic characteristics (age, socioeconomic status, education level, grandmother's year of schooling) were assessed at time of recruitment into the study (8‐16 weeks gestation). Symptoms of maternal prenatal depression and anxiety were also assessed at baseline using the Brazilian Portuguese versions of the Beck Depression and Anxiety Inventories (BDI, BAI; Beck & Cunha, 2001). Family food insecurity was measured with a brief version of the Brazilian Food Insecurity Scale (Santos et al., 2014). Trained psychologists administered the scales to mothers in interview format.

#### 2.4.2 Assessment of secondary outcome measures

##### Mother‐infant attachment relationships at infant age 12 months

A video‐recorded 14‐min mother‐infant interaction was conducted when the infants were aged 12 months. The interaction was semi‐structured and included five situations (playing, teaching, helping, setting boundaries, dealing with stress and separation) to elicit attachment behaviors (see eAppendix 2). Videos were coded using the Emotional Availability Scale (EAS, Biringen, 2008), a widely used instrument to assess parent‐child interactions that shows good reliability and validity in varied populations (Biringen et al., 2014; Ziv et al., 2000). The EAS quantifies emotional attachment between the parent and child in six dimensions: (1) Caregiver Sensitivity (affective presence/appropriate responsiveness to child), (2) Caregiver Structuring (use of proper strategies to guide child/set preventative limits), (3) Caregiver Non‐intrusiveness (ability to withhold behaviors that interfere with child's interests and support age‐appropriate autonomy), (4) Caregiver Non‐hostility (absence of subtle/overt negative emotions), (5) Child Responsiveness to the caregiver (responsiveness to parent's communication bids), and (6) Child Involvement with the caregiver (the extent to which child invites the parent to interact with them).

EAS measures of attachment relationships used in the current study were *(1) Direct scores*, which quantify the quality in each of the six dimensions (scored 1‐to‐7 with higher scores indicative of better attachment behavior, see eTable 2) and *(2) Emotional Attachment Zones* computed using the *Emotional Attachment & Emotional Availability Clinical Screener (EA‐CS*; Biringen, 2008). The EA‐CS uses the Caregiver Sensitivity and Child Responsiveness dimensions to quantify caregiver‐child emotional attachment and assign parents/children to one of four attachment zones (*Emotionally Available*, *Complicated*, *Detached*, and *Problematic/Disturbed*), which correspond to four attachment styles (*Secure, Insecure Anxious‐Resistant, Insecure‐Avoidant*, and *Disorganized*, Ainsworth et al., 1978; Main & Solomon, 1986; see eTable 2). The proportion of mothers and infants in each group in the Emotionally Available Attachment Zone was used as a measure of secure attachment in the current analysis. The EAS‐CS is well‐validated and correlates highly with independent measures of mother‐child attachment behavior (Zero to Three Task Force, 2005; Ziv et al., 2000). One coder and the first author blinded to randomization were trained in EAS coding and certified by the EAS developer (Z Biringen). Each rater independently assessed all mother‐infant dyads’ interactions. Inter‐rater reliability was high for all six EAS dimensions (Intra*ICC* > .992). The first author coding was used in analyses.

**TABLE 2:** Summary of secondary outcome measures: attachment measures at age 12 months and neural correlates of social development at age 6 months by group

	**CAU**	**Intervention**
**Age 12‐month assessment of mother‐infant attachment**		
*Sample size for analysis (% female infants)*	28 (39.3%)	28 (53.6%)
*Maternal age (years; mean, SD)*	18.81 (1.54)	18.65 (1.56)
*Infant age (weeks; mean, SD)*	56.74 (4.67)	57.69 (6.05)
*EAS Direct scores (mean, SD, 95% CI)*:		
*EAS Maternal sensitivity*	4.61 (1.50) (4.03‐5.19)	4.86 (1.48) (4.28‐5.43)
*EAS Maternal Structuring*	3.79 (1.51) (3.20‐4.37)	3.95 (1.44) (3.39‐4.50)
*EAS Maternal Non‐Intrusiveness*	5.14 (1.30) (4.64‐5.65)	5.16 (1.63) (4.52‐5.80)
*EAS Maternal Non‐Hostility*	5.50 (1.30) (5.00‐6.00)	5.54 (1.50) (4.96‐6.12)
*EAS Child Responsiveness*	4.75 (1.24) (4.27‐5.23)	5.31 (1.43) (4.75‐5.86)
*EAS Child Involvement*	3.66 (1.52) (3.07‐4.25)	4.55 (1.46) (3.99‐5.12)
*EAS Maternal Emotional Attachment Zone (N, %)*:		
*Emotionally available*	11 (39.3%)	13 (46.4%)
*Complicated*	10 (35.7%)	9 (32.1%)
*Detached*	7 (25.0%)	5 (17.9%)
*Problematic/disturbed*	0	1 (3.6%)
*EAS Infant Emotional Attachment Zone (N, %)*:		
*Emotionally available*	10 (35.7%)	18 (64.3%)
*Complicated*	12 (42.8%)	5 (17.9%)
*Detached*	6 (21.4%)	5 (17.9%)
*Problematic/disturbed*	0	0
		
**Age 6‐month assessment of neural correlates of infant social development**		
*Sample size for analysis (% female infants)*	13 (46.2%)	15 (53.3%)
*Infant age (weeks; mean, SD)*	26.62 (1.26)	27.07 (1.17)
*No. of clean EEG trials in analysis (mean, SD, min‐max)*:		
*Mother condition*	41.08 (17.25, 15–65)	39.20 (14.76, 15–63)
*Stranger condition*	41.23 (17.16, 15–66)	40.47 (16.10, 16–66)
*Mean Nc amplitude (μv) (mean, SD)*:		
*Mother condition – frontal scalp*	−3.54 (4.33)	−0.05 (5.22)
*Stranger condition – frontal scalp*	−2.46 (4.71)	−1.69 (4.06)
*Mother condition – central scalp*	−5.53 (3.06)	−5.84 (5.26)
*Stranger condition – central scalp*	−6.36 (6.04)	−5.36 (3.32)

*EAS,  *Emotional Availability Scale; *Mean Nc amplitude*,  the mean amplitude measured in the time‐range of the Nc (400‐600 ms); *μv, *amplitude measured in microvolts.

##### Neural correlates of infant social development at infant age 6 months

At age 6 months, infants completed a passive viewing task during EEG recording while seated on their mother's lap ∼65 cm in front of a 45 cm × 45 cm screen in a dimly lit room. The task presented static images (11 × 18 cm) of the mother's or a stranger's (another study mother's) face. Task trials began with a 1200 ms pause (blank screen) followed by a central fixation cross (black on a white background) presented until the infant was looking at the screen (timing controlled by the experimenter) and then the mother or stranger face image for 500 ms and finally an inter‐trial‐interval of 1200 ms (blank screen). Seventy‐five images of the mother and 75 images of the stranger were presented in randomized order (see de Haan & Nelson, 1997).

One photo of the mother and one photo of the stranger was used for each baby and the two photos were repeated multiple times. Members of the research team conducting the assessments took the photos of the mothers on the day the babies took part in the EEG assessment. All mothers stood in the same location in the laboratory against a blank white wall and wore a cream‐colored cloak that covered their clothes while their photograph was taken. Mothers were asked to relax their faces and maintain a neutral expression, while looking directly at the camera. The researchers then cropped the photos to be the same size and number of pixels and to ensure only the head and shoulders were included in the frame before the photos were added to the experimental programming software that was used to present the task. For each baby, the mother's photo was matched with a photo from one of the other mothers in the study who had a similar appearance in terms of hairstyle, facial features, skin coloring, and use of glasses.

EEG data were recorded using a 128‐channel Geodesic Sensor Net and a NetAmp 200 DC‐coupled amplifier (Electrical Geodesics Inc., Oregon, USA). A video‐recording of the baby was simultaneously collected and synchronized with the EEG data. The data were referenced online to electrode Cz, sampled at 500 Hz and bandpass filtered between 0.1‐100 Hz. EEG data were processed offline using Brain Vision Analyzer v2.1 (Brain Products, Munich, Germany) by the second author blinded to randomization. The video‐recording of each baby was reviewed and periods in which the baby looked away from the screen, had their eyes closed or was crying were marked in the EEG data; these sections of the EEG data were excluded from further processing and analysis. Electrodes around the rim of the net were contaminated by the excessive artifact and removed from all participants, leaving 80 electrodes in the analysis. The data were filtered using 0.1 Hz high‐pass, 30 Hz low‐pass 24 dB/Oct Butterworth filters with a 60 Hz notch filter for residual electrical line noise. Periods of data with excessive noise were excluded. Flat or noisy channels were removed and interpolated using spherical spline interpolation before re‐referencing to the average reference and removing ocular artifacts using independent components analysis. The data were segmented into −200 to +1000 ms stimulus‐locked epochs for the mother and stranger conditions separately. Epochs with remaining artifacts (amplitudes +/−150 μv) were excluded. Clean epochs were baseline‐corrected using the −200‐0 ms period and averaged to create ERPs for the mother and stranger conditions. Infants with fewer than 15 artifact‐free epochs in either condition were excluded from the analysis.

The Nc was measured as the mean amplitude (μv) in the 400–600 ms post‐stimulus time‐range at frontal and central electrode clusters. The frontal cluster included Fz and the surrounding five electrodes; the central cluster included Cz and the surrounding five electrodes (see eFigure 1). Mean amplitude of the Nc was extracted for each condition (mother/stranger) and cluster (frontal/central).

##### Infant temperament at age 12 months

Infant temperament was measured using the Brazilian Portuguese version of the Infant Behavior Questionnaire‐Revised (IBQ‐R, Gartstein & Rothbart, 2003) administered to mothers in interview format by trained psychologists. The IBQ‐R yields scores for three temperament factors: Surgency (approach and positive affect), Negative Affect (fear, sadness, distress) and Regulation (longer attending/better orienting to objects, higher soothability). These temperament factors were included in analysis as a covariate since child temperament is proposed to influence mother‐infant attachment (Bowlby, 1969).

### 2.5 Statistical analyses

Statistical analyses were conducted in STATA 15 (Stata, version 15.1; StataCorp). Group differences in baseline characteristics were assessed with independent‐samples t‐tests (or Mann‐Whitney U tests for non‐normally distributed variables) for continuous variables and Chi‐square tests for categorical variables.

To test the hypothesis that mother‐infant dyads who received the intervention would show stronger attachment relationships than those who received CAU (*hypothesis 1*), linear and logistic regression models were conducted with intervention group predicting EAS Direct scores (six linear models) and the proportion of mothers and infants categorized as being in the EAS Emotionally Available Attachment Zone (two logistic models). Eight models were conducted in total to test *Hypothesis 1*. Unadjusted and adjusted odds ratios (OR), unstandardized beta coefficients (B), 95% confidence intervals, and *p*‐values are reported. Adjusted models included infant temperament factors (Surgency, Negative Affect, Regulation) as continuous independent variables.

To test the hypothesis that infants in the intervention group would show more differentiated neural responses to mother and stranger face stimuli compared to those in the CAU group (*hypothesis 2*), one mixed‐model ANOVA was conducted on mean Nc amplitudes with the between‐subjects factor group (intervention, CAU) and the within‐subjects factors electrode cluster (frontal, central) and condition (mother, stranger). Significant interactions between the factors were further investigated using Bonferroni‐corrected planned pairwise contrasts between the levels of each factor. The ANOVA was repeated with the covariates age (given known effects of age on the Nc, Carver et al., 2003; de Haan & Nelson, 1997) and infant temperament factors.

To test the hypothesis that more differentiated neural correlates of social development at age 6 months would be associated with better social‐interaction behavior with mothers at age 12 months (*hypothesis 3*), Spearman correlation coefficients were computed between 6‐month mean Nc amplitudes for mother/stranger conditions and dimensional 12‐month mother/infant EAS Direct scores. To limit the number of tests conducted, correlation coefficients were only computed for mother/infant EAS Direct scores that differed significantly between groups. One multinomial logistic regression model was conducted to test whether 6‐month mean Nc amplitudes to mother/stranger faces at frontal and electrode clusters predicted 12‐month Emotionally Available Attachment Zones.

## 3 RESULTS

### 3.1 Sample characteristics

Mothers from the intervention and CAU groups did not differ significantly in baseline sociodemographic characteristics (Table 1). Mothers in the intervention group received a mean of 27.7 (SD = 14.2) home‐visits by the 12‐month attachment assessment. At the age 12‐month assessment of attachment relationships, 56 mother‐infant dyads (28 interventions, 28 CAU) provided usable data for analysis. At the age 6‐month assessment of neural correlates of attachment development, 28 infants (15 interventions, 13 CAU) provided usable data for analysis. Fifty infants (25 interventions, 25 CAU) completed EEG recording. Data from 14 infants were unusable due to technical problems with the EEG system. A further eight infants had fewer than 15 artifact‐free epochs for analysis and were also excluded, leaving a final sample for analysis of *n *= 28 (15 intervention, 13 CAU) infants. Retention analysis revealed no differences in variables that may have introduced bias between participants with and without secondary outcome data (see eAppendix 2).

### 3.2 Mother‐infant attachment relationships at infant age 12 months

EAS Direct scores and EAS Emotional Attachment Zones are shown by group in Table 2. Results of the linear and logistic regression models testing effects of intervention group on these variables are presented in Table 3. Linear regression showed that membership of the Intervention compared to CAU group was associated with significantly higher Child Involvement scores, but was not significantly associated with the remaining EAS Direct scores. Logistic regression revealed a significant effect of intervention group on Child Emotionally Available Attachment Zone, with a significantly higher proportion of infants classified as Emotionally Available (securely attached) in the intervention than CAU group. Group did not predict maternal Emotionally Available Attachment Zone.

**TABLE 3:** Results of linear and logistic regression models testing the effects of the intervention on mother‐infant attachment relationships when infants were aged 12 months

	**Unadjusted models**			**Adjusted models**		
*EAS Direct scores*	B	95% CIs	*p* value	B	95% CIs	*p* value
*Maternal Sensitivity*	0.3	−0.4, 1.2	0.383	0.4	−0.4, 1.2	0.335
*Maternal Structuring*	0.3	−0.5, 1.1	0.498	0.3	−0.4, 1.2	0.382
*Maternal Non‐Intrusiveness*	−0.03	−0.3, 0.2	0.814	−0.01	−0.3, 0.2	0.904
*Maternal Non‐Hostility*	0.1	−0.7, 0.9	0.801	0.1	−0.7, 0.9	0.745
*Child Responsiveness*	0.5	−0.2, 1.3	0.136	0.6	−0.1, 0.4	0.086
*Child Involvement*	0.9	0.1, 1.7	0.023*	1.0	0.1, 1.8	0.022*
***EAS Emotional Attachment Zones***	**OR**	**95% CIs**	***P* value**	**OR**	**95% CIs**	***P* value**
*Maternal Emotionally Available Zone*	1.6	0.5, 4.9	0.379	1.7	0.5, 5.3	0.356
*Child Emotionally Available Zone*	3.4	1.1, 10.4	0.032*	4.7	1.3, 16.8	0.016*

^a^ Adjusted models included infant temperament factors (Surgency, Negative Affect, Regulation) measured at age 12 months as a covariate. Asterisks (*) highlight significant model results.

### 3.3 Neural correlates of infant social development at infant age 6 months

**FIGURE 2:**
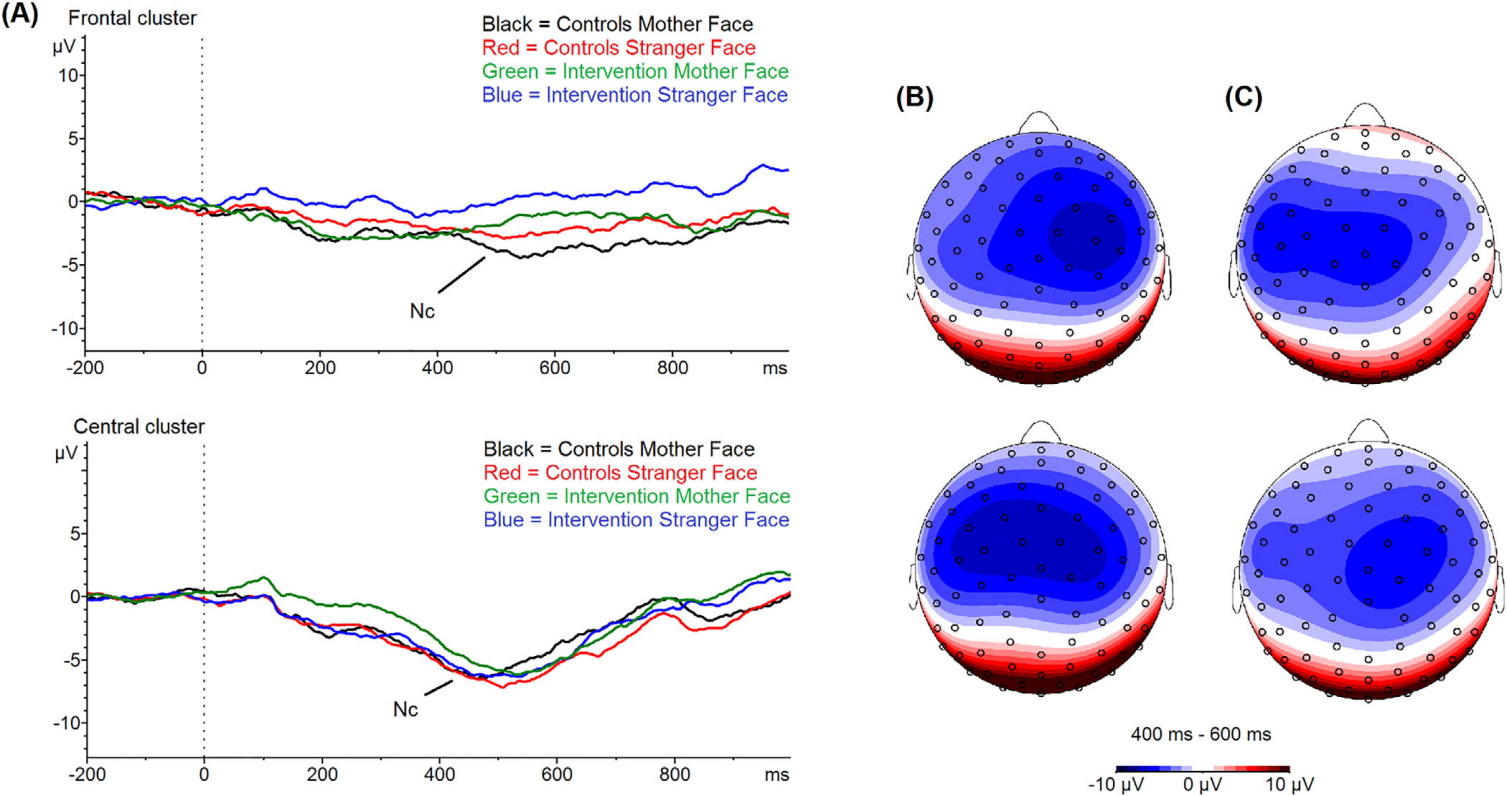
Grand average waveforms and topographical plots of the Nc ERP component by condition and group. Grand averaged waveforms (A) are plotted by group and condition at the frontal (top) and central (bottom) electrode clusters with the Nc component indicated. Topographies of the Nc are shown for (B) control (CAU) infants and (C) intervention infants in the mother (top) and stranger (bottom) conditions

Grand averages of the Nc are plotted by condition, group, and electrode cluster in Figure 2; group means are shown in Table 2. The 2 × 2 × 2 ANOVA revealed no significant effect of group on mean Nc amplitudes (F(1,26) = 1.69, *p* = 0.205, η^2 ^= 0.06) and no significant interactions between group and electrode cluster or condition (all F ≤ 1.42, *p* ≥ 0.244).

### 3.4 Associations between neural correlates of infant social development at age 6 months and mother‐infant attachment relationships at age 12 months

**FIGURE 3:**
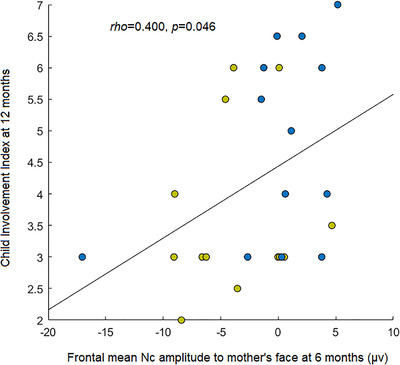
Associations between neural correlates of social development at 6 months and attachment behavior at 12 months. Scatterplot shows the significant positive correlation between mean amplitude of the Nc component while viewing the mother's face at frontal scalp at age 6 months and infants' attachment behavior (EAS child involvement index) at 12 months. The regression line represents the correlation computed in the intervention and CAU groups combined; for information purposes only, intervention and CAU infants are plotted in different colors (blue = intervention, yellow = control)

Spearman correlation coefficients were computed between mean Nc amplitudes for the mother and stranger faces at the frontal and central electrode clusters and the EAS Direct score variables that differed significantly between groups (Child Involvement Index scores; four correlation coefficients were computed in total). Mean Nc amplitude at the frontal cluster in the mother condition was significantly positively correlated with EAS Child Involvement index (rho(26) = .400, p = .046); infants with more positive (smaller) Nc amplitudes had higher involvement scores (Figure 3). There were no further significant associations (rho ≤ .300, *p* ≥ .126). The multinomial logistic regression model examining whether mean Nc amplitude for mother/stranger conditions at frontal and central electrodes predicted child emotional attachment zone was non‐significant (χ^2^(8) = 14.65, *p* = 066).

## 4 DISCUSSION

Consistent with our hypothesis, the *Primeiros Laços* HVP had significant positive effects in promoting secure attachment relationships at age 12 months, indicated by higher child involvement scores and a larger proportion of infants classified as in an emotionally available attachment zone. Strikingly, rates of secure attachment in infants who received care‐as‐usual (35.7%) were comparable to those reported in previous studies of infants of adolescent mothers (e.g., 33% in Moran et al., 2008), while rates of secure attachment in infants whose mothers received the intervention (64.3%) were similar to those reported in samples of typically developing infants (e.g., 59% in Lewis‐Morrarty et al., 2014; 67% in Bakerman‐Kranenburg & van IJzendoorn, 2009). These findings suggest that *Primeiros Laço*s buffers the adverse effects of risk factors such as poverty and adolescent motherhood on early attachment development. These positive effects likely have long‐term benefits. Behaviors quantified by the EAS Child Involvement index (e.g., making social overtures to the mother) are early indicators of good social interaction skills, which are protective factors contributing to resilience (Domitrovich et al., 2017). Secure early attachment relationships are protective against a range of psychological, socio‐emotional and behavioral problems in children growing up in adversity (Groh et al., 2017). Further, early‐life attachment behaviors are transmitted across generations (Verhage et al., 2016). Therefore, early HVP interventions such as *Primeiros Laços* represent important methods of breaking transgenerational insecure attachment cycles in high‐risk families.

However, in contrast to our hypothesis, there were no effects of the intervention on maternal attachment behaviors. Previous work has suggested that attachment relationships depend on the sensitivity of the caregiver's responsiveness to the needs of the child (Zeegers et al., 2017) and that maternal sensitivity is more amenable to intervention than infant attachment security (Bakermans‐Kranenburg et al., 2003). Contrary to this work, *Primeiros Laços* did not affect maternal attachment dimensions, including maternal sensitivity. Still, previous intervention studies targeted at attachment in maltreated infants have similarly indicated that maternal sensitivity did not underlie positive effects of interventions on infant attachment (Cicchetti et al., 2006). Thus, maternal sensitivity may not play as crucial a role as was believed in improving infant attachment development, at least in high‐risk populations. The question then remains of which factors were involved in the improvement in infant attachment in our study. One possible factor is infant temperament; previous research has shown that both infant temperament and parental sensitivity influence the development of secure mother‐infant attachment relationships (Groh et al., 2017; Zeeger et al., 2017). However, adjusting for infant temperament in our analyses did not alter the findings, indicating that intervention‐related improvements in infant attachment were independent of infant temperament. Another possible factor is the increased attention infants received from the home‐visitor. The *Primeiros Laços* nurses talked to the baby and reflected the baby's emotions to promote emotional processing and regulation while modeling maternal behavior. These regular interactions with the nurses may have had therapeutic effects for the infants and contributed to infants' attachment behaviors. Indeed, the relationship between the home‐visitor and the family is central to the success of such interventions (Saïas et al., 2016).

The positive effect of *Primeiros Laços* on infant attachment behaviors at 12 months was supported by the pattern of findings in neural indices of infant social development at age 6 months. In contrast to our hypotheses and previous work (de Haan & Nelson, 1997), the intervention group did not show more differentiated Nc responses to mother and stranger faces compared to CAU infants. However, our dimensional analysis revealed that smaller Nc amplitudes to mother stimuli were correlated with better child involvement scores 6 months later. This finding is consistent with previous reports of significant correlations between lower Nc amplitude to mother face stimuli and increased interaction‐seeking behaviors with the mother in 6‐month‐old infants of adult mothers (Swingler et al., 2007) as well as lower Nc amplitudes to the mother's face in securely vs. insecurely attached preschoolers (Kungl et al., 2017).

Larger Nc amplitudes are indicative of greater attentional allocation to salient stimuli (de Haan & Nelson, 1997). Reduced Nc amplitudes in association with increased infant social behavior has been interpreted in terms of reduced attentional allocation to the mother because the infant has already established that the mother is the person to whom they should direct behavior, i.e. the foundation of the attachment bond has already been formed, allowing infants to consolidate that relationship via interaction‐seeking behaviors while employing less effortful attentional resources to the mother (Swingler et al., 2007). To our knowledge we are the first to report that Nc amplitude reductions predict infant social behavior longitudinally and that this association is present in infants of impoverished adolescent mothers in LMICs who are vulnerable to adverse developmental outcomes. Indeed, while many authors have emphasized the importance of early social relationships in guiding infant brain development and protecting against negative effects of adverse early environments on child development (Newman et al., 2015), how human infant attachment and neurobiology are related in the first months of life has rarely been investigated (for one exception see Tharner et al., 2011).

Our Nc findings may be informative as to the mechanisms by which *Primeiros Laços* improves infant attachment development. Fewer attentional resources devoted principally to the mother (smaller Nc amplitudes) may lead to better social‐interaction skills in the first year of life and underpin the development of secure attachment in vulnerable infants. It will be important for future work to identify which aspects of *Primeiros Laços*, such as the relationship with the home‐visitor or additional social stimulation for infants, drive these changes in infants’ neurobehavioral development.

Our findings should be interpreted in the context of several limitations. Our sample size was modest, particularly for the EEG measures, and attachment data were collected at only one time‐point. Future work in larger samples should include longitudinal measures of both neural correlates and behavioral measures of mother‐infant attachment to better understand the effects of HVPs on the developmental trajectory of attachment in vulnerable mother‐infant dyads. Future studies should also examine factors mediating the impact of HVPs on mother‐infant attachment, including maternal psychopathology, which has been negatively associated with secure mother‐infant attachment relationships (Verhage et al., 2016). We did not measure the abilities of the home‐visitors or their relationships with the family; these factors should be investigated in further research to assess the extent to which they determine the effectiveness of HVPs on early attachment development.

## 5 CONCLUSIONS

This study is the first to demonstrate that an HVP grounded in attachment theory can enhance the early development of attachment in infants of adolescent mothers living in poverty in Brazil. Furthermore, our findings suggest that earlier‐life enhancements in neural circuitry involved in social processing may contribute to improvements in infant attachment development. Our findings indicate that HVPs could be important tools for improving early socio‐emotional development of vulnerable infants in low‐resource countries.

## CONFLICTS OF INTEREST

Guilherme V. Polanczyk has been in the past 3 years a consultant member of the advisory board and/or speaker for Shire/Takeda and Medice. He received travel expenses for continuing education support from Shire/Takeda and royalties from Editora Manole. The other authors report no conflicts of interest.

## FUNDING

This study was funded by grants from Grand Challenges Canada (GCC), Fundação Maria Cecília Souto Vidigal (0722‐03), Bill & Melinda Gates Foundation (OPP1142172), Conselho Nacional de Desenvolvimento Científico e Tecnológico (420267/2016‐6), National Institute of Developmental Psychiatry for Children and Adolescents (INPD), São Paulo Research Foundation (FAPESP, refs 2014/50917‐0 and 2016/22455‐8), CNPq (465550/2014‐2) and Companhia Brasileira de Metalurgia e Mineração. Elizabeth Shephard is supported by a postdoctoral fellowship from the São Paulo Research Foundation (FAPESP, ref 2018/22396‐7).

## Supporting information

Supporting Information

## Data Availability

The data that support the findings from this study are not publicly available due to confidentiality and ethical reasons. The data can be requested from the corresponding author, Professor Guilherme V Polanczyk (gvp@usp.br).
