# Supplementary material for: Promoting mother‐infant relationships and underlying neural correlates: Results from a randomized controlled trial of a home‐visiting program for adolescent mothers in Brazil
Source: Dev Sci. 2021 Apr 12;24(6):e13113. doi: 10.1111/desc.13113 (PMC8596406; doi:10.1111/desc.13113)
Supplement: Supplementary file 1 — Supporting Information [file DESC-24-e13113-s001.docx]

**Supplementary Material**

Alarcão et al. Promoting Mother-Infant Relationships and underlying neural correlates: Results from a Randomized Controlled Trial of a Home-Visiting Program for Adolescent Mothers in Brazil

**eTable 1. Axes, Objectives and Interventions of Primeiros Laços**

**eAppendix 1. Assessment of mother-infant interaction**

**eTable 2. Emotional Availability Scale and corresponding attachment classification**

**eFigure 1. Electrode map showing electrode clusters used for measurement of the Nc**

**eAppendix 2. Retention analysis**

**eTable 1. Axes, Objectives and Interventions of Primeiros Laços**

| ***Axis*** | ***Objective*** | ***Intervention(s)*** |
| --- | --- | --- |
| *Health care* | Provide support and stimulate the mother to take care of her and her child’s health | Appropriate nutrition; avoid consumption of tobacco, alcohol and other drugs; personal hygiene; instructions and educational materials on prevalent diseases; immunization; accidents |
| *Health environment* | Provide support for the mother in identifying, understanding and implementing concepts that create a good environment for each phase of the child’s development | Adequate and safe housing; access to education and health resources; community support |
| *Life project* | Help the mother to identify her personal life goals | Stimulate and reinforce other social roles (e.g. continuing study, building a family, searching for jobs) driven by the mothers’ observed needs and reported interests |
| *Parenting and attachment* | Promote healthy parent-child relationships based on socio-emotional, cognitive and language development | Emphasize warm, responsive, reflective, structuring, non-intrusive and non-hostile parenting skills; reinforce family strengths; cognitive stimulation |
| *Family and social network* | Ensure access to public services is available to support mother and infant’s needs | Map social network of the family (existing informal sources e.g. neighbours, community resources, barriers to networking) |

**eAppendix 1. Assessment of mother-infant interaction**

*1. Free play (4 minutes)*
Mother and child are asked to play together as they normally would. A box of toys is available if they want to explore. The purpose is to observe the mother’s ability to be warm, responsive and to follow the child’s leads and interests. In addition, the researcher shows a wrapped package, places it one meter away from the child and tells the mother that the child cannot open it during the free play. When the 4-minute period is finished, the researcher asks the mother to unwrap the package. The purpose is to observe if the mother sets limits, controls the child and teaches self-regulation. How the child interacts, whether the child invites the adult to play with them and how he/she exercises his/her autonomy to explore the environment is also observed.

*2. Mother helps child to complete a puzzle (4 minutes)*

The mother is asked to help the child to complete a puzzle that was in the wrapped package. The purpose is to observe how the mother tries to teach the child including whether she uses verbal and non-verbal communication to structure and what strategies she uses under pressure. In addition, how the child attends, collaborates and exercises his/her autonomy is observed.

*3. Separation and reunion (1 minute)*

The mother is asked to leave the room and stay outside for 1 minute before returning. The purpose is to observe child attachment behaviours.

*4. Tidy toys (3 minutes)*

The mother is asked to instruct the child to put the toys in the box. The purpose is to observe if the mother can get the child to cooperate and if she mother is fun, flexible and creative. How the child attends and receives input and how he/she exercises his/her autonomy is also observed.

*5. Bubble play (2 minutes)*

The mother is asked to play with the child and to try to teach the child how to produce soap bubbles. The purpose is to observe how the mother tries to teach, if she uses verbal and non-verbal communication to structure, and what strategies she uses under pressure. How the child interacts, if he/she invites the mother to join in his/her play, and if he/she learns/accepts suggestions and guidance are also observed.

**eTable 2. Emotional Availability Scale^1,2^ and corresponding attachment classification**

| **Emotional Attachment/emotional availability (EAS)** | **Attachment Classification (Ainsworth)** |
| --- | --- |
| Emotionally Available | Secure |
| Complicated | Insecure anxious-resistant |
| Detached | Insecure avoidant |
| Problematic/Disturbed | Disorganized |

^1^ Biringen Z et al. Emotional availability (EA): Theoretical background, empirical research using the EA Scales, and clinical applications. Developmental Review (2014).

^2^ Saunders, H et al. Emotional Availability and Emotional Availability Zones (EA-Z): From assessment to intervention and universal prevention. Perspectives in infant mental health (2017).

**eFigure 1. Electrode map**
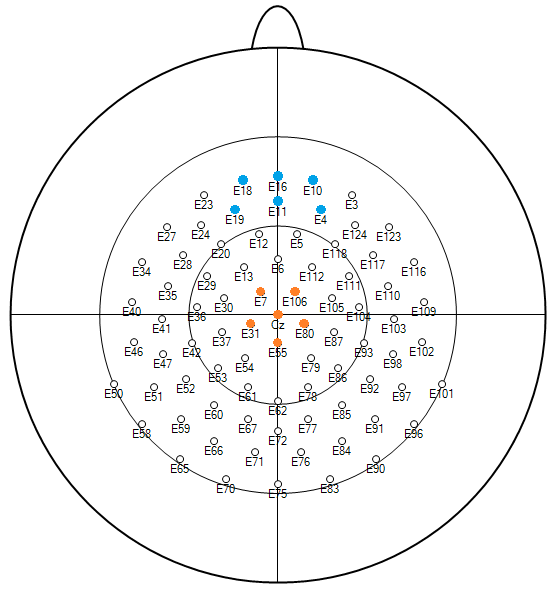


Electrodes included in the frontal (blue) and central (orange) electrode clusters for measurement of the Nc component

**eAppendix 2. Retention analysis**

Retention analyses were conducted to assess whether the mother-infant dyads who did and did not provided usable attachment and infant temperament data at 12 months differed from each other on variables that may have introduced bias in the analyses, specifically maternal age, infant age, infant temperament and maternal symptoms of anxiety and depression. Mother-infant dyads who did (EA+, n=54) and did not (EA-, n=24) provide usable attachment and infant temperament data did not differ in any of the specified characteristics: maternal age at baseline (mean = 17.05, sd = 1.38; EA- mean = 17.23, sd = 0.95; *t*(78) = -0.58, p= .56), maternal depression at baseline (EA+ mean = 12.94, sd = 8.14; EA- mean = 11.11, sd = 7.33; *t*(78) = 0.97, p= .0.33), maternal anxiety at baseline (EA+ mean = 11.00, sd = 7.53; EA- mean = 9.19, sd = 7.21; *t*(78) = 1.02, p= .31), infant age at the age 12-month assessment (EA+ mean = 10.38, sd = 4.84; EA- mean = 10.76, sd = 5.38; *t*(63) = -0.23, p= .0.82), Surgency at age 12 months (EA+ mean =5.12, sd = 0.84; EA- mean = 5.20, sd = 1.01; *t*(60) = -0.23, p= .82), Negative Affect at age 12 months (EA+ mean =4.05, sd = 0.84; EA- mean = 4.18, sd = 0.60; *t*(60) = -.0.43, p= .67), and Regulation at age 12 months (EA+ mean = 4.96, sd = 0.97; EA- mean = 4.80, sd = 0.79; *t*(60) = 0.43, p= .67).

Retention analyses were also conducted to determine whether infants who did and did not provide usable EEG data at age 6 months differed from each other on variables (age, temperament, maternal symptoms of depression and anxiety) that may have influenced the findings. Due to technical problems with the EEG system, data could only be collected from 50 infants at age 6 months (25 intervention, 25 care-as-usual). Among the 50 infants with whom EEG was attempted, data from 14 infants were unusable due to technical problems with the EEG system. A further eight infants had fewer than 15 artefact-free epochs for analysis and were also excluded, leaving a final sample for analysis of *n*=28 (15 intervention, 13 CAU) infants. Infants who did (EEG+, n=28) and did not (EEG-, n=52) provide usable EEG data for analysis did not differ in age at the 6-month-old EEG assessment (EEG+ mean = 5.96, sd = 0.33; EEG- mean = 5.95, sd = 0.22; (*t*(67) = -0.20, p = .84), temperament factors Surgency (EEG+ mean = 5.17, sd = 0.86; EEG- mean = 5.11, sd = 0.85; *t*(60) = -.024, p = .81), Negative Affect (EEG+ mean = 3.87, sd = 0.81; EEG- mean = 4.22, sd = 0.78; *t*(60) = 1.74, p = .09) or Regulation (EEG+ mean = 4.78, sd = 0.99; EEG- mean = 5.06, sd = 0.90; *t*(60) = 1.15, p = .25) measured at age 12 months, maternal depression at baseline (EEG+ mean = 13.25, sd = 8.42; EEG- mean = 11.87, sd = 7.62; *t*(78) = -0.75, p = .46), maternal anxiety at baseline (EEG+ mean = 10.54, sd = 7.50; EEG- mean = 10.35, sd = 7.47; *t*(78) = -0.11, p = .91) or maternal age (years) at baseline (EEG+ mean = 16.93, sd = 1.36; EEG- mean = 17.21, sd = 1.19; *t*(78) = 0.96, p = .34).
